# Improved Prognostic Prediction in Never-Smoker Lung Cancer Patients by Integration of a Systemic Inflammation Marker with Tumor Immune Contexture Analysis

**DOI:** 10.3390/cancers12071828

**Published:** 2020-07-07

**Authors:** Massimo Milione, Mattia Boeri, Anna Cantarutti, Giovanni Centonze, Adele Busico, Paola Suatoni, Giovanna Garzone, Laura Cattaneo, Elena Tamborini, Federica Perrone, Alessandro Mangogna, Giovanni Corrao, Giancarlo Pruneri, Gabriella Sozzi, Andrea Anichini, Ugo Pastorino

**Affiliations:** 1 Department of Pathology and Laboratory Medicine, Fondazione IRCCS Istituto Nazionale dei Tumori, via Venezian 1, 20133 Milan, Italy; giovanni.centonze@istitutotumori.mi.it (G.C.); adele.busico@istitutotumori.mi.it (A.B.); giovanna.garzone@istitutotumori.mi.it (G.G.); laura.cattaneo@istitutotumori.mi.it (L.C.); elena.tamborini@istitutotumori.mi.it (E.T.); federica.perrone@istitutotumori.mi.it (F.P.); giancarlo.pruneri@istitutotumori.mi.it (G.P.); 2 Tumour Genomics Unit, Department of Research, Fondazione IRCCS Istituto Nazionale Tumori, via Venezian 1, 20133 Milan, Italy; mattia.boeri@istitutotumori.mi.it (M.B.); gabriella.sozzi@istitutotumori.mi.it (G.S.); 3 Division of Biostatistics, Epidemiology and Public Health, Department of Statistics and Quantitative Methods, University of Milano-Bicocca, 20126 Milan, Italy; anna.cantarutti@unimib.it (A.C.); giovanni.corrao@unimib.it (G.C.); 4 Thoracic Surgery Unit, Department of Surgery, Fondazione IRCCS Istituto Nazionale Tumori, via Venezian 1, 20133 Milan, Italy; paola.suatoni@istitutotumori.mi.it (P.S.); ugo.pastorino@istitutotumori.mi.it (U.P.); 5 Pathology Unit, Clinical Department of Medical, Surgical and Health Sciences, University of Trieste, Ospedale di Cattinara, Strada di Fiume 447, 34149 Trieste, Italy; alessandro.mangogna@studenti.units.it; 6 Human Tumors Immunobiology Unit, Department of Research, Fondazione IRCCS Istituto Nazionale Tumori, via Venezian 1, 20133 Milan, Italy; andrea.anichini@istitutotumori.mi.it

**Keywords:** never-smokers, inflammation, immune profile

## Abstract

Almost 25% of lung cancers (LCs) occur in never-smokers. LC inflammatory profile, based on plasma C-reactive protein levels (CRP), predicts mortality, independently by smoking-status. We hypothesized that: CRP could be associated with tumor immune contexture (TIC) in never-smokers and both these two parameters may improve their prognosis. Sixty-eight never-smokers LC patients with high or low CRP were selected. The programmed cell death protein 1 (PD-1) and its ligand (PD-L1), the human leukocyte antigens (HLA-DR and HLA-I), CD8, CD4, CD3, CD33, CD163, and CD68 were evaluated by immunohistochemistry on surgical samples given TIC evaluation. The classification model based on TIC scores was generated by Classification and Regression Tree analysis. Tumor mutational burden was evaluated by targeted next-generation sequencing. Exclusively high CRP (H-CRP) subset showed PD-L1 expression in 35% of LC as well as lower HLA-I and HLA-DR in their stromal cells. CD3, CD4, CD8, HLA-I, HLA-DR tumor cells staining were associated with a “low inflammatory profile” subset. CRP and LC immune profiles drive clinical outcome: 5-year survival 88% against 8% was associated with low and high-risk profiles (*p* < 0.0001). Clinical outcome prediction in never-smoker LC patients may be improved by both CRP and tumor immune contexture evaluation.

## 1. Introduction

Despite tobacco consumption remaining the main cause of lung cancer worldwide [1], it is estimated that almost one-fourth of all lung cancer (LC) occurs in never-smokers, reaching over 50% in women [2]. Of these, adenocarcinoma accounts for about 80% of cases in never-smokers and up to 77% are detected in advanced stages (IIIB-IV), thus making surgery unfeasible for the majority of these patients [3]. Among patients with the operable disease, no differences in terms of overall survival (OS) were observed between never- and ever-smokers [4].

Compared with ever-smokers, tumors of never-smokers LC patients appear to be less immunogenic [5], with less infiltrating CD8 T cells [6], fewer neoplastic cells expressing programmed death-ligand 1 (PD-L1) and lower tumor mutational burden (TMB) [7,8]. On the other hand, molecular analysis revealed that mutations involving *EGFR*, *BRAF*, *ALK-EML4*, and *ROS1* genes are more frequent in never than in current or former smokers [9]. Indeed, presence of targetable mutations were found in up to 73% of never-smokers LC patients [3,10], thus making these tumors more responsive to tyrosine kinase inhibitors (TKIs) rather than to immune checkpoint inhibitors (ICIs) [11,12,13].

Circulating inflammation markers such as interleukins (IL) and C-reactive proteins (CRP) have been widely associated with survival in LC patients [14,15,16]. However, these studies included ever-smoker LC patients with altered immune and inflammation markers and were not powered to analyze differences among subsets of never-smokers LC patients [17].

As a consequence of this scenario, tumor-promoting inflammation and immune-escape mechanisms were among the hallmarks of cancer less analyzed in never-smokers LC patients [18]. We have recently reported that the inflammatory profile based on CRP values at baseline (CRP_0_) and 3 days after surgery (CRP_3_) predict mortality in a consecutive series of 1750 operable LC patients, irrespective of smoking status [19]. Indeed, even if CRP_0_ and CRP_3_ levels maintained an independent prognostic value, a synergic effect was observed in patients where both CRP measurements exceeded respective cut-offs (CRP_0_ > 3 and CRP_3_ > 126 mg/L) [19].

In the present study, the programmed cell death protein 1 (PD-1) and its ligand (PD-L1), the human leukocyte antigens (HLA-DR and HLA-I), CD3, CD8, CD4, characterizing T cells, as well as myeloid markers such as CD33, CD163, and CD68 were evaluated by immunohistochemistry (IHC) on surgical samples collected from 68 operable never-smokers LC patients with high and low CRP plasma levels. In order to identify additional microenvironment features characterizing the tumor tissue and improving prognostic assessment, a semi-quantitative score system, taking into account staining marker extent and intensity within tumor cells (reported using suffix “T”, i.e. PD-1_T), intra- (suffix “I”, i.e. PD-1_I) and extra-tumor (suffix “E”, i.e. PD-1_E) immune content, was adopted.

## 2. Results

### 2.1. Clinico-Pathological Characteristics According to Patients Inflammatory Profile

From a consecutive series of 1750 operable LC patients, 68 never-smokers patients with high or low inflammatory profiles given by CRP levels were selected with a 1:1 matching design for age, gender, and tumor stage (Table 1). The two groups of patients had similar characteristics for non-matching variables such as tumor histology (*p* = 0.1092) and body mass index (BMI) values (*p* = 0.3486). Conversely, a better lung functionality as measured by forced expiratory volume in the first second of expiration (FEV_1_) (*p* = 0.0070) and less death events at both two (*p* < 0.0001) and five (*p* = 0.0006) years were observed in patients with low inflammatory profiles (Table 1).

### 2.2. Tumor Immune Features Are Associated with the Systemic Inflammatory Profile

**Figure 1:**
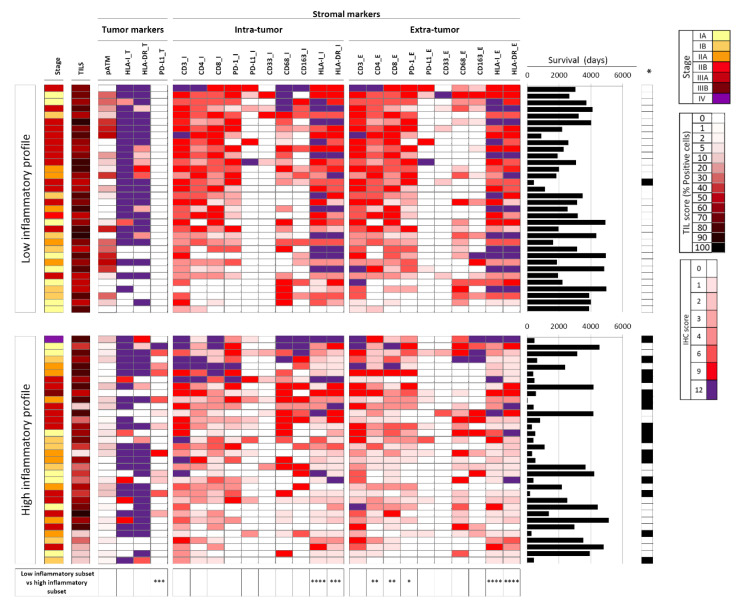
Sixty-eight lung cancer (LC) patients stratified according to the inflammation profile: high vs. low. The expression of each marker was evaluated within tumor cells (by adding “_T” to marker name) or in the stroma intra-tumor (_I) and extra-tumor component (_E). On the right, for each patient a graph is shown indicating length of patient survival (days) and related death/censoring information (*Black: dead of disease; white: censored). Differentially expressed markers in the high vs low inflammatory profiles are indicated by the asterisks at the bottom of the figure: 1, 2, 3, or 4 asterisks indicated Mann Whitney test *p*-values *p* < 0.05, *p* < 0.01, *p* < 0.001, or *p* < 0.0001, respectively.

By comparing immunohistochemistry (IHC) scores on neoplastic cells of patients with high and low inflammatory profile, a PD-L1_T positive signal was found in 12 patients, all with high inflammatory profile (35% of this subset, *p* = 0.0002, Figure 1). Both HLA-I_T and HLA-DR_T were highly expressed in both groups of patients. On the other hand, HLA-I_I (*p* < 0.0001), HLA-DR_I (*p* = 0.0002), HLA-I_E (*p* < 0.0001), and HLA-DR_E (*p* < 0.0001) were significantly less expressed in stromal cells of patients with high inflammatory profile. Despite significance being reached only on the extra-tumor immune component, a reduction of CD4 (*p* = 0.0066), CD8 (*p* = 0.0061), and PD-1 (*p* = 0.0196) signals on stromal cells of patients with high inflammatory profile was also observed (Figure 1). Representative images of IHC signal were reported in Figure S1. All the other markers, including the percentages of tumor-infiltrating lymphocytes (TILs), did not differ when comparing the two groups of patients.

In order to gain insight on the relationships of all immune markers in the two groups of lesions, we used Spearman correlation analysis. First, each marker evaluated in the intra-tumor compartment showed a significant and positive association with the same molecule evaluated in the extra-tumor compartment, in both high and low inflammatory profile subsets (Figure S2A,B). However, several significant differences were also found in the two groups with high vs low inflammatory profile. An inverse correlation was found between HLA-I/DR on tumor and the same markers evaluated on intra or extra-tumor stromal cells, but almost exclusively in the high inflammatory subset (Figure S2B). On the other hand, a positive associations of CD3_I/E, CD4_I/E, CD8_I/E with HLA-I_T, and HLA-DR_T was observed almost uniquely in the low inflammatory profile group (Figure S2A).

To further support the hypothesis of an association between peripheral/systemic marker of inflammation (CRP) with the overall structure of the tumor microenvironment, we carried out Spearman correlation analysis testing the association of CRP_0_, CRP_3_, and CRP_max_ values with any of the tumor immune markers. Results in the whole set of 68 lesions indicated significant and inverse correlations of all three CRP values with CD3_E, CD4_I/E, CD8_E, PD-1_E, with HLA-I_I/E and HLA-DR_I/E, but a striking positive association with PD-L1_T (Figure S3). In the high inflammatory subset, CRP_0_ was positively associated with CD163_E (a potential marker of pro-tumoral macrophages) and negatively with CD8_I. Additional negative associations were found between CRP_3_ or CRP_max_ with CD3_E or CD4_E or PD-1_E (Figure S3).

### 2.3. Combining Immune and Inflammatory Features to Improve the Prognostic Value

**Figure 2:**
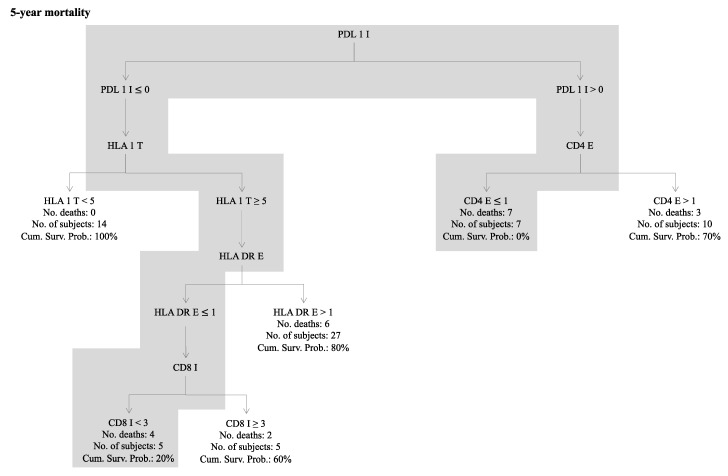
The classification tree model based on immune scores to classifying patients according to their 5-year risk of death. The gray line identifies patients with high immune profile and worse 5-year mortality.

A prognostic decision tree model based on immune scores was generated considering the whole series and the 5-year mortality as end-point (Figure 2). Two groups of patients were considered as “high risk immune profile”: the first composed by 5 subjects characterized by null PD-L1_I expression, HLA-I_T ≥ 5, HLA-DR_E ≤ 1, and CD8_I < 3 resulting in a 20% of cumulative survival probability; while the second was composed by 7 patients, who died within 5 years, with positive PD-L1_I expression and CD4_E ≤ 1. All these 12 immune high-risk patients also had a high inflammatory profile. In agreement, a “low risk immune profile” was defined as any lesion showing the opposite profile for both criteria.

**Figure 3:**
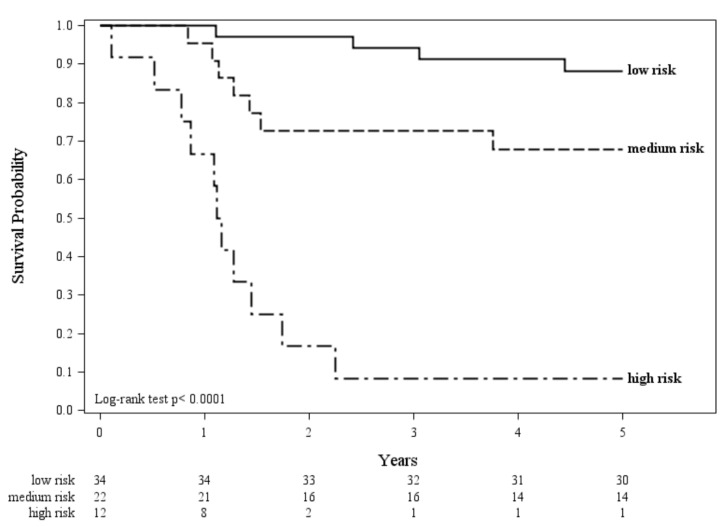
5-year overall survival of LC patients stratified into three groups according to the inflammatory and the immune profile: low inflammatory and low immune profile (low risk), high inflammatory and low immune profile (medium risk), high inflammatory and high immune profile (high risk). Log-rank test *p*-value is reported.

By combining inflammatory and immune profile, patients were thus stratified in low (both low inflammatory and low risk immune profile), medium (high inflammatory profile but low risk immune profile), and high risk (both high inflammatory and high risk immune profile) with 5-year overall survival of 88%, 68%, and 8%, respectively (Figure 3, *p* < 0.0001). Overall, an increased risk of 5-year mortality for patients with high inflammatory profile (hazard ratio (HR): 6.8; 95% confidence interval (CI): 2.3–20.2) and patients with high risk immune profile (HR: 12.1, 95% CI: 4.9–28.5) taken individually (model 1 and 2) was found (Table 2). In the multivariable Cox model (model 3), the immune profile, but not the inflammatory profile, was still significant. Additionally, the combined observed effect reported in model 4 (HR: 22.5; 95% CI: 6.8–74.4) corresponded to the expected value according to model 3 (expected HR: 21.5). Taken together, these results suggest that prognosis in never-smoker LC patients is at least in part determined by interaction of a systemic effect (high or low inflammatory profile) and of a tumor microenvironment feature (high or low risk immune profile). All models were adjusted for tumor histology.

### 2.4. Tumor Mutational Status Across Immune/Inflammatory Profiles

In a subset of 42 patients with adequate tumor tissue specimens, targeted next generation sequencing (NGS) was also performed. Overall, 280 missense mutations, 34 trunc, and 16 in-frame were found in 185 genes. *EGFR* was the most frequently mutated gene (31%), followed by *TP53* (29%), *SYNE1* (17%) and *ERBB2* (14%) (Figure S4). Neither single mutation, nor the TMB was associated with the inflammatory profile. On the other hand, presence of mutations in the *ATM* gene were associated with the high-risk immune profile (*p* = 0.0113). When considering the single immune markers, patients harboring *EGFR* mutations were characterized by a lower T lymphocyte content (Figure S5) in terms of both CD3_I (*p* = 0.0297) and CD3_E (*p* = 0.0023).

## 3. Discussion

The role of inflammation and the immune system in the development and treatment of lung cancer has gained increasing attention over the last decade. Cigarette smoking is among the main factors associated with lung cancer and has been correlated with higher levels of circulating CRP and differences in tumor immune cell composition [17]. Lower level of resting mast cells and resting CD4 memory T cells as well as higher levels of the activated counterparts characterize the tumor microenvironment in ever-smokers [5]. Nevertheless, information on the inflammatory status and an immune characterization of never-smokers LC patients is still lacking. Here we investigated the molecular characteristics of tumor tissue and its immune microenvironment in resectable never-smoker LC patients with high and low plasma CRP levels. The limited sample size should be however acknowledged when interpreting the present results.

As previously described in larger consecutive series including current and former-smokers [19,20], pre- and post-operative CRP levels have a prognostic role even when considering only never-smokers lung cancer patients. Analysis of the tumor tissue of these patients revealed that PD-L1 positive tumors were exclusively in the high CRP group with worse prognosis. In the same group, a reduction of HLA-I and HLA-DR was also observed in both intra- and extra-tumor stromal cells. Interestingly, despite both being significantly deregulated in patients with high inflammatory profiles, PD-L1_T and HLA expression on stromal cells were not associated. These data point out that the expression of PD-L1 by tumor cells and presence of surrounding immune cells with a lower ability to present tumor antigens for CD8 and CD4 T cell activation could be considered complementary mechanisms to evade immune surveillance in presence of high systemic inflammation levels. In addition, the reduction of the CD8 and CD4 stromal signal, reaching significance in the extra-tumor immune component, further indicate that these tumors recruit less T lymphocytes.

Exclusively in the high inflammatory subset, an inverse correlation takes place between the recruitment of HLA-DR+ stromal cells, such as macrophages, dendritic cells, B cells, activated T cells, and expression of HLA-DR on neoplastic cells. On the other hand, given the positive association between the presence of lymphoid markers (CD3, CD4, and CD8) and HLA tumor expression, only the low inflammatory subset seems to have the ability to develop an effective T cell infiltrate whenever HLA antigen expression on tumor cells is retained. Taken together these results suggest that a peripheral marker of inflammation (CRP_0_, CRP_3_, and CRP_max_), evaluated at time of surgery, is associated with/predicts the existence of a compromised immune contexture in the tumor microenvironment.

The different immune landscapes observed in neoplastic lesions from patients belonging to the low vs. high inflammatory subsets may hint at a further mechanism: the immune landscape of the high CRP subset may be skewed towards a pro-tumoral profile. Such immune landscape may be characterized by production of cytokines that in turn activate of the acute phase protein response at the systemic level. In this potential scenario, high CRP levels may be considered as the systemic response to a pro-inflammatory, pro-tumoral condition developing at tumor site.

Among the 28 immune biomarkers, PD-L1_I, HLA-I_T, HLA-DR_E, CD8_I, and CD4_E contributed to classifying patients according to their 5-year risk of death in low or high immune risk profile. By fitting Cox models for the 5-year overall survival adjusting for both the inflammatory and the immune profile, the inflammatory profile lost its prognostic value, thus suggesting a dependent effect between the two profiles. Moreover, there seems to be a multiplicative effect of inflammatory and immune profile, since when grouping patients with both high risk profiles we observed an HR = 21.5 compared to patients with both low risk profiles.

Tumor intrinsic markers, such as HLA-I or the tumor mutational status, were not associated with the systemic inflammatory profiles given by CRP levels. Conversely, patients with *EGFR* mutations where characterized by lower CD3 T lymphocytes infiltrating and surrounding tumor cells, while mutations in the *ATM* gene were associated with the high immune risk profile. These data strengthen the evidence of a correlation between tumor specific mutations and its immune microenvironment [21,22].

## 4. Materials and Methods 

### 4.1. Study Population and Data Collection

Data were collected from Fondazione IRCCS Istituto Nazionale dei Tumori in Milan, a referral oncological Center in Northern Italy, from January 2003 to December 2015. The cohort included non-smokers patients who underwent their first resection for primary LC at the Thoracic Surgery Division. Collected baseline data, recorded in a dedicated database of our Institution, included demographics (age and gender), lifestyle (smoking), anthropometric (BMI), respiratory functionality (percent predicted—FEV1), and tumor characteristics (stage and histology).

This study was conducted in compliance with the Declaration of Helsinki and approved by the review board of Fondazione IRCCS Istituto Nazionale dei Tumori of Milan (protocol number: INT 171/16). Written informed consent was obtained from the patients.

The plasma level of CRP measured at baseline (CRP_0_) three days (CRP_3_) after surgery as well as the maximum value observed in the postoperative course (CRP_max_) were recorded as previously described [19]. Cases where both pre/post-surgery CRP levels were below a pre-specified threshold (CRP_0_ ≤ 3 mg/l and CRP_3_ ≤ 126 mg/L) were classified as showing a low inflammatory profile, while patients with CRP_0_> 3 mg/L and CRP_3_ > 126 mg/L as showing a high inflammatory profile [19]. For each patient with high inflammatory profile, one patient with low inflammatory profile was randomly selected to be matched for stage of disease, gender, and age. Data on biomarkers of immune profile were also collected for all patients. The latter included: (i) inhibitory receptors and ligands (PD-1, PD-L1), (ii) HLA molecules (HLA-DR and HLA-I), (iii) T cell markers (CD3, CD4, CD8), (iv) myeloid markers (CD33, CD163 and CD68), (v) TMB, DNA damage (pATM), and (vi) tumor-infiltrating lymphocytes. All markers detected by IHC were evaluated on tumor cells (when appropriate, such as HLA-I, HLA-DR, PD-L1) as well as on intra-and extra-tumor stroma. Patients were followed until the date of death or November 21, 2018 for survivors.

### 4.2. Immunohistochemistry (IHC) Analysis

IHC analysis on formalin-fixed paraffin-embedded (FFPE) tumor tissue sections were carried out using appropriate positive and negative controls for each antibody. Consecutive sections were stained with antibodies directed to CD3, CD4, CD8, PD-1, PDL-1, CD33, CD68, CD163, HLA-I, and HLA-DR markers as described [23]. A semi-quantitative score system was adopted taking into account both staining marker extent (% positive cells) and intensity within tumor cells, intra- and extra-tumor immune content [23]. The extension score ranged from 0 (0% of positive cells), to 1 (up to 25% of positive cells), 2 (26–50% positive cells), 3 (51–75% positive cells), until 4 (76–100% positive cells). The intensity of the immunostaining of each marker was compared and ranked in comparison with the signal of internal controls: 1 if fainter, 2 if equal, or 3 if more intense. The product of extension x intensity were combined into a single score, as previously described [23].

### 4.3. Targeted Next Generation Sequencing

Tumor DNA was extracted from manually microdissected 5-µm FFPE sections using the QIAamp Gene Read DNA FFPE kit (Qiagen, Hilden, Germany). Targeted NGS was performed using Oncomine Tumor Mutational Load Assay (Thermo Fisher, Waltham, MA, USA) covering 1.65 megabases of 409 genes with known cancer associations according to the manufacturer’s instructions. Data were processed using the Ion Torrent Suite software (version 5.8.0) and the Variant Caller plugin as previously described [24]. The Oncomine TML workflow 5.10 on Ion reporter software was adopted to calculate the TMB, considering only nonsynonymous somatic variants not included in the 1000 Genome Project, 5000Exomes Global, and ExAC.

### 4.4. Statistical Analysis

Paired parametric (t test), no parametric (Wilcoxon signed-rank), and McNemar tests were used where appropriate for comparing biological parameters of LC patients. Association of all immune markers and of immune markers with CRP_0_, CRP_3_, and CRP_max_ values was investigated through spearman correlation analysis. To identify the immune biomarker combinations predictive of five-year mortality a classification tree model was used. The Classification and Regression Tree (CART) analysis was used to generate a simple and practical clinical decision rule [25]. Every value of each considered biomarker is considered as a potential split, and the CART method divides a selected range of variables to obtain an optimal binary split into two subgroups. Impurity criterion is adopted as the node splitting rule. From this, CART analysis generates a classification tree and numerical rank for each input used to build the tree by relative importance. We grouped patients into two risk levels (high- and low-risk immune profile) based on the risk of death in each group [26,27]. We defined two mutually exclusive risk profiles based on the following criteria: a “high risk immune profile” is any lesion that fits at least one of the two following criteria: 1) PD-L1 = 0, HLA-I_T ≥ 5, HLA-DR_E ≤ 1 and CD8_I < 3. 2) PD-L1 > 0, CD4_E < 1. A low risk immune profile is any lesion that fits the opposite profile for the same criteria.

The Kaplan Meier estimator was used to evaluate overall survival according to the combination of inflammatory and immune profile. Patients were categorized as low risk (i.e., having both low inflammatory and low risk immune profile), medium risk (i.e., having a high inflammatory profile but a low risk immune profile), or high risk (i.e., having both high inflammatory and high risk immune profile). Time-to event comparisons were made using log-rank test. The prognostic value of both inflammatory and immune profile in predicting two- and five-year mortality was investigated by fitting Cox’s proportional hazard regression models. The effects of the inflammatory and immune profile were investigated alone and together. All analyses were performed using the Statistical Analysis System Software (version 9.4; SAS Institute, Cary, NC, USA) and the RStudio (version 1.3.959; R studio, Boston, MA, USA). Statistical significance was set at the 0.05 level. All *p*-values were two-sided.

## 5. Conclusions

To our knowledge, this is the first study comparing the tumor tissue and its immune microenvironment with different systemic inflammatory profiles in never-smokers LC patients. Indeed, our data indicate that higher levels of inflammation correspond to a more immunocompromised tumor microenvironment characterized by low HLA levels and less T lymphocytes. Moreover, prognosis determination is improved by integrating the two parameters. This hypothesis should be further investigated in dedicated studies.

## Figures and Tables

**Table 1:** Selected characteristics of study patients.

Patients Characteristics	Low Inflammatory Profile		High Inflammatory Profile		*p*-Value
	(N = 34)		(N = 34)		
At baseline					
*Matching variables*					
Age - mean (SD)	66	(9.7)	67	(12.2)	0.7761
Female Gender - nr. (%)	22	(64.7)	20	(58.8)	0.9064
Stage of disease - nr. (%)					0.8353
I	14	(41.2)	13	(38.2)	
II	6	(17.7)	8	(23.5)	
III-IV	14	(41.2)	13	(38.2)	
*No-matching variables*					
FEV_1_ (%) - median (IQ range)	96.5	(50)	74	(47)	0.0070
BMI - median (IQ range)	24.5	(5)	25	(3)	0.3486
During follow-up					
Two-year mortality					
deaths - nr. (%)	1	(2.9)	16	(47.1)	<0.0001
person-years - mean (SD)	1.9	(0.2)	1.6	(0.5)	
mortality rate - x100 person-years	1.5		29.9		
Five-year mortality					
deaths - nr. (%)	4	(11.8)	18	(52.3)	0.0006
person-years - mean (SD)	4.7	(0.8)	3	(1.9)	
mortality rate - x100 person-years	2.5		17.8		

Notes: SD, standard deviation; nr., number; IQ, inter-quartile; FEV_1_, forced expiratory volume in the first second of expiration; BMI, body mass index.

**Table 2:** Relationship between Inflammatory profile, immune profile, and risk of death at 5 years.

	Model 1	Model 2	Model 3	Model 4
	HR (95% CI) *	HR (95% CI) *	HR (95% CI) *	HR (95% CI) *
High inflammatory profile	6.8 (2.3–20.2)		3.4 (1.0–11.8)	
High risk immune profile		12.1 (4.9–28.5)	6.6 (2.5–17.6)	
Inflammatory and immune profile				
Low risk				1.0 (reference)
Medium risk				3.4 (1.0–11.7)
High risk				22.5 (6.8–74.4)

*Adjusted for tumor histology.

## Supplementary Materials

The following are available online at https://www.mdpi.com/2072-6694/12/7/1828/s1, Figure S1: Representative immunohistochemical features in tumor tissue (A,C,E,G) and its microenvironment (B,D,F,H). PD-L1 staining was observed on the membrane of tumor cells (A), but not in stromal cells (B). Conversely, PD-1 signal was absent in neoplastic (C) and positive in stromal cells (D). In neoplastic cells, HLA-1 is extensively expressed in the cytoplasm (E), while HLA-DR is only focally expressed (G). Both HLA-1 and HLA-DR signals are well defined and extensively located within the tumor microenvironment (F-H), Figure S2: Heat map analysis reporting the Spearman correlation data of all possible comparisons of immune scores of all markers in LC patients with (A) low and (B) high inflammatory profile. Color coding of r and *p*-values: green indicates a positive correlation, while yellow-to-reds indicates a negative correlation. Color intensity is proportional to *p* value, see legend, Figure S3: Spearman correlation analysis for association of all immune markers evaluated in the tumor lesions by IHC with the CRP_0_, CRP_3_ and CRP_max_ values in the whole set of patients (*n* = 68) and in the two subsets defined by low and high inflammatory profile. Positive and negative r values are highlighted in green and red respectively. Only significant correlations are shown, Figure S4: Mutation plot of 42 lung tumors resected from never-smokers lung cancer patients. Co-mutation plot of genes mutated in at least 3 samples and clinico-pathological characteristics of patients. Genes are ranked according to the mutation frequency. Samples are ranked according to the tumor mutational burden (TMB) in the three groups of patients: low inflammatory and low immune profile; high inflammatory and low immune profile; and high inflammatory and high immune profile, Figure S5: Distribution of CD3 IHC score in intra- and extra-tumor stromal cells in patients with *EGFR* mutated (mut) and *EGFR* wild type (wt).
