# Supplementary material for: Improved Prognostic Prediction in Never-Smoker Lung Cancer Patients by Integration of a Systemic Inflammation Marker with Tumor Immune Contexture Analysis
Source: Cancers (Basel). 2020 Jul 7;12(7):1828. doi: 10.3390/cancers12071828 (PMC7408913; doi:10.3390/cancers12071828)
Supplement: Supplementary file 1 [file cancers-12-01828-s001.docx]

**Supplementary Materials**


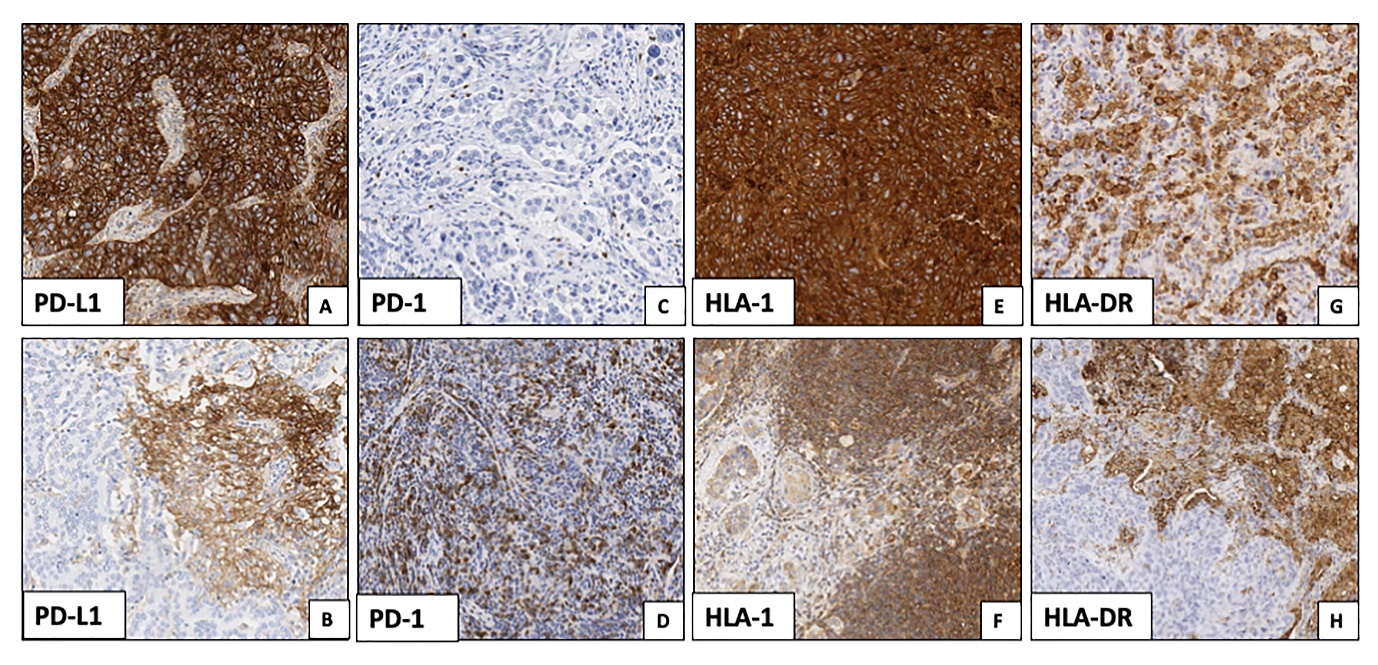


**Figure S1.** Representative immunohistochemical features in tumor tissue (A,C,E,G) and its microenvironment (B,D,F,H). PD-L1 staining was observed on the membrane of tumor cells (**A**), but not in stromal cells (**B**). Conversely, PD-1 signal was absent in neoplastic (**C**) and positive in stromal cells (**D**). In neoplastic cells, HLA-1 is extensively expressed in the cytoplasm (**E**), while HLA-DR is only focally expressed (**G**). Both HLA-1 and HLA-DR signals are well defined and extensively located within the tumor microenvironment (**F–H**).


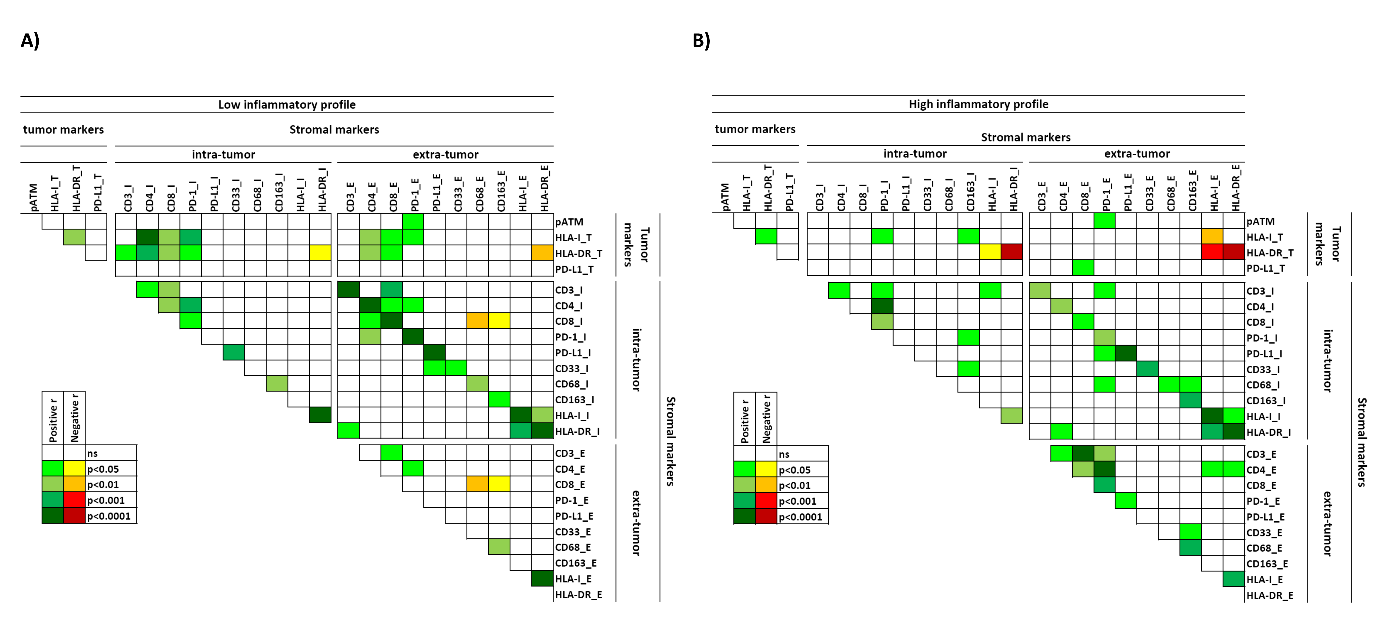


**Figure S2.** Heat map analysis reporting the Spearman correlation data of all possible comparisons of immune scores of all markers in LC patients with (**A**) low and (**B**) high inflammatory profile. Color coding of *r* and *p*-values: green indicates a positive correlation, while yellow-to-reds indicates a negative correlation. Color intensity is proportional to *p*-value, see legend.


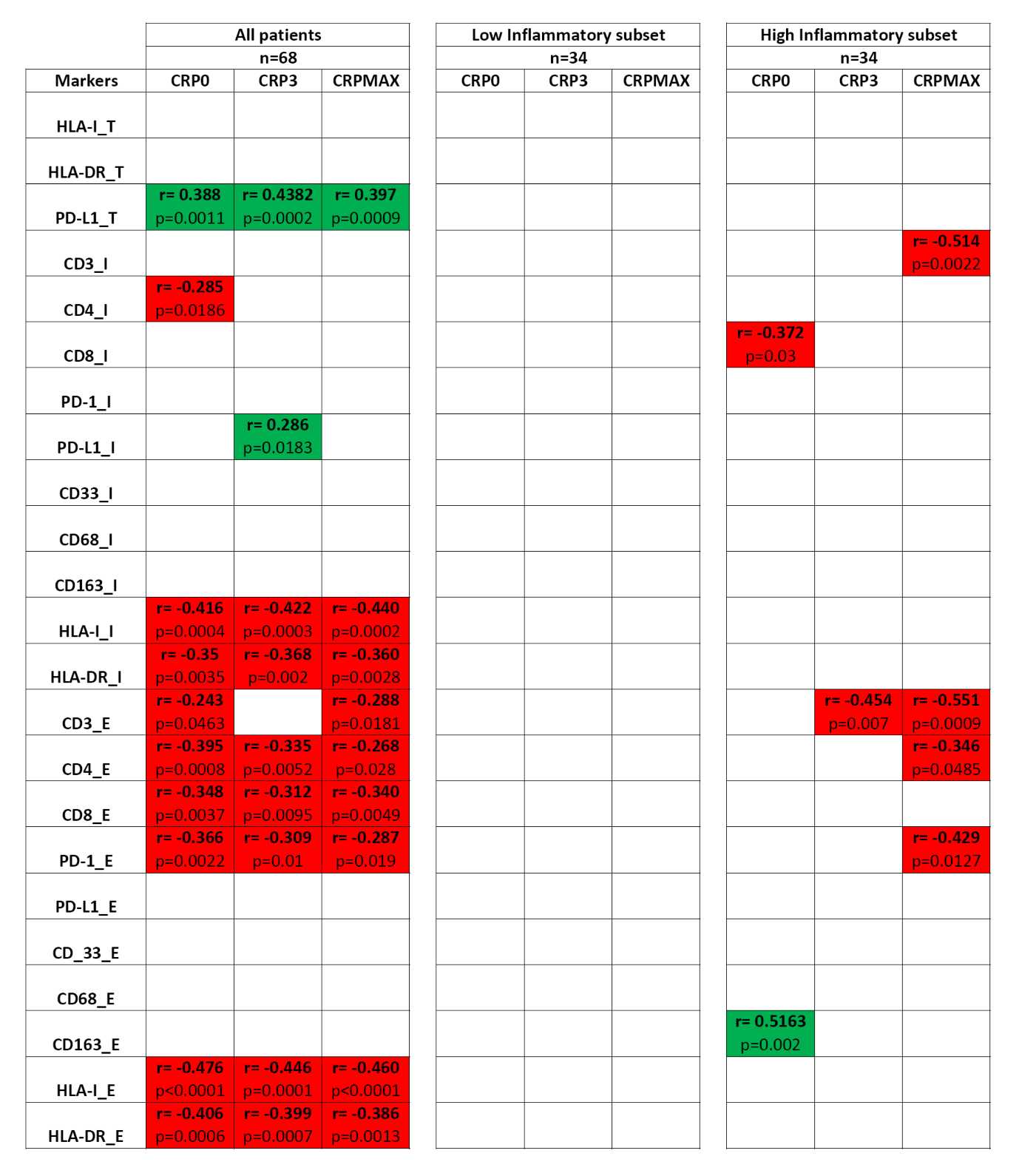


**Figure S3.** Spearman correlation analysis for association of all immune markers evaluated in the tumor lesions by IHC with the CRP_0_, CRP_3_ and CRP_max_ values in the whole set of patients (*n* = 68) and in the two subsets defined by low and high inflammatory profile. Positive and negative *r* values are highlighted in green and red respectively. Only significant correlations are shown.


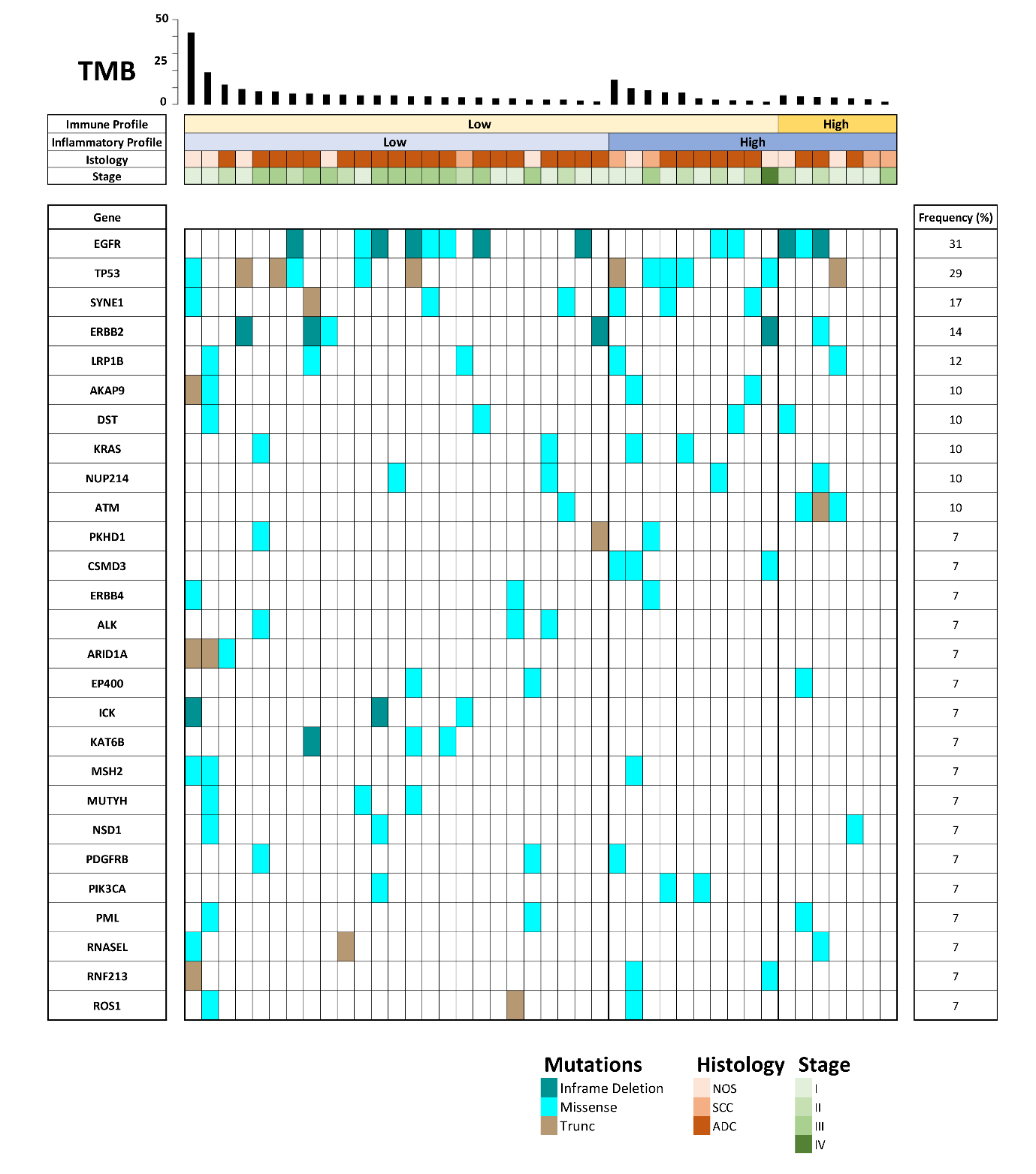


**Figure S4.** Mutation plot of 42 lung tumors resected from never-smokers lung cancer patients. Co-mutation plot of genes mutated in at least 3 samples and clinico-pathological characteristics of patients. Genes are ranked according to the mutation frequency. Samples are ranked according to the tumor mutational burden (TMB) in the three groups of patients: low inflammatory and low immune profile; high inflammatory and low immune profile; and high inflammatory and high immune profile.


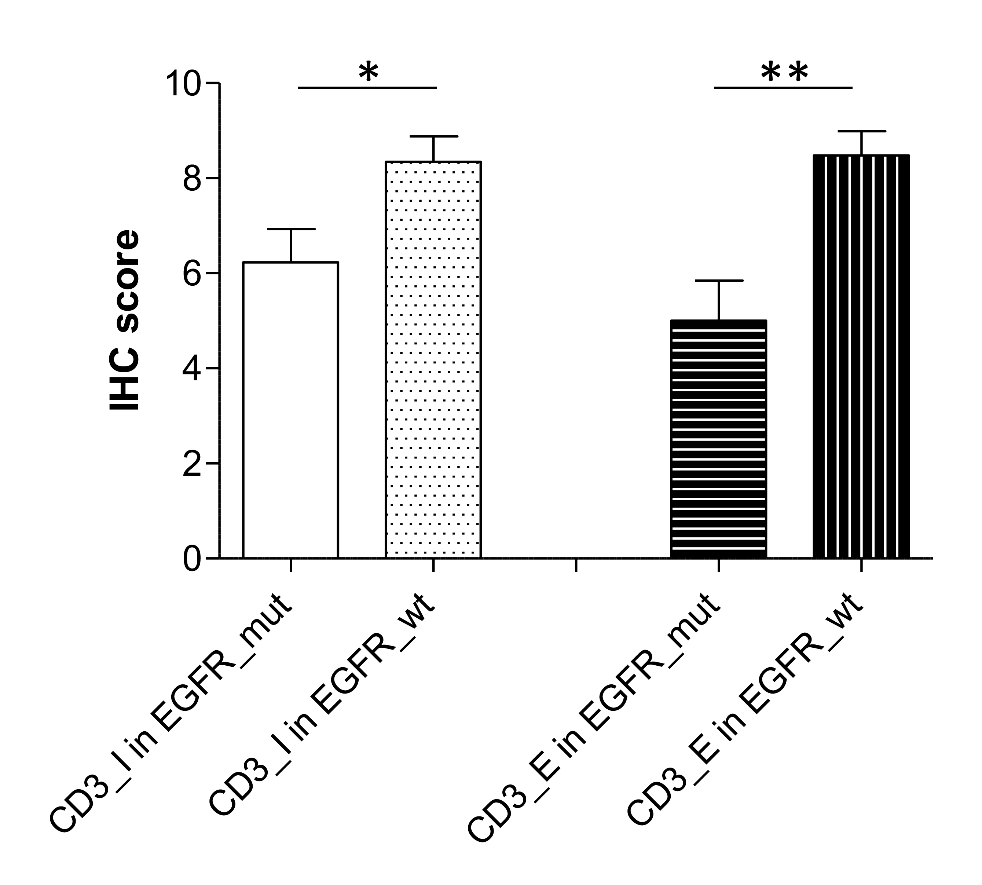


**Figure S5.** Distribution of CD3 IHC score in intra- and extra-tumor stromal cells in patients with *EGFR* mutated (mut) and *EGFR* wild type (wt). * and ** indicate *p*-values *p* < 0.05, *p* < 0.01, respectively
